# Assessment of Urban Flood Vulnerability Using the Integrated Framework and Process Analysis: A Case from Nanjing, China

**DOI:** 10.3390/ijerph192416595

**Published:** 2022-12-10

**Authors:** Peng Wang, Yifan Zhu, Ping Yu

**Affiliations:** Faculty of Civil Engineering and Mechanics, Jiangsu University, Zhenjiang 212013, China

**Keywords:** flood, urban flood vulnerability assessment, contribution analysis, process analysis

## Abstract

Flooding is a serious challenge that increasingly affects residents as well as policymakers. Many studies have noted that decreasing the urban flood vulnerability (UFV) is an indispensable strategy for reducing flood risks; however, some studies have several pertinent assessment limitations. The objective of this study is to assess the UFV of the Xuanwu-Qinhuai-Jianye-Gulou-Yuhua (XQJGY) region from 2012 to 2018 by integrating various indicators into a composite index. This study uses the environment for visualizing images (ENVI) and the geographic information system (GIS) to extract indicators that have geographic attributes for the assessment of UFV and the process analysis method is then used to explore the relationship between these indicators. The results indicated that: (1) The UFV of Xuanwu, Qinhuai, and Gulou decreased from 2012 to 2018 and the UFV of Jianye and Gulou increased from 2012 to 2015 and decreased from 2015 to 2018. (2) The vegetation coverage, precipitation during the flood season, population density, and highway density significantly contributed to the UFV. (3) There also exist transformation pathways between the indicators that led to vulnerability in five districts. This study provides a theoretical basis for the government to manage floods.

## 1. Introduction

In recent decades, extreme weather events and meteorological disasters have occurred frequently in the context of global warming [1,2]. Urban flooding is a major natural global hazard that is becoming increasingly frequent and serious in the world [3,4,5]. Currently, more than half of the world’s population lives in urban areas [6]. As a result of floods, 19,000 people are killed, 12,000 people are injured, and 150,000 people lose their homes per year all over the world [7]. Therefore, it is necessary and urgent to adopt strategies for adapting to flooding, which suggests decision makers must utilize flood prevention measures in advance to reduce economic losses and casualties.

Vulnerability estimation is an indispensable aspect of a flood risk assessment. With the acceleration of urbanization processes, urban physical forms have changed dramatically, increasing the uncertainties in the vulnerability assessment process. For example, built-up land can drive flood hazard dynamics by altering hydrological and hydraulic processes [8]. From 1992 to 2015, the Chinese built-up land area increased by 542% (or 26 × 103 km^2^) [9]. Therefore, approaches to assess the urban flood vulnerability (UFV) should consider urban physical forms. Vulnerability to urban floods have been evaluated in different countries and the aim of a flood vulnerability assessment is to provide theoretical support for decision makers to develop flood mitigation strategies. Most of these studies have remained at the evaluation level and used multi-dimensional variables, including physical, economic, community, nature, population, and political variables. In addition, these studies have explored the risk levels of urban floods for populations in specific places. Nonetheless, the internal drive mechanisms of the chosen variables have not been widely reported. The internal drive mechanisms of the chosen variables provide theoretical support for decision makers to develop mitigation strategies.

The overall purpose of this study is to develop an integrated urban flood vulnerability assessment approach that can accurately assess the UFV and explore the internal drive mechanisms of the chosen indicators. Using the case of the XQJGY region, the aim is to (1) measure the spatial and temporal characteristics of the study region; (2) assess the contribution of each index to the UFV in the study region; (3) investigate the drive mechanism pathways between the indicators; and (4) develop strategies for mitigation and adaptation to flooding.

### 1.1. Literature Review

Vulnerability has become a central focus for global environmental change science research communities in recent years [10]. Vulnerability is a complex concept with different characteristics in the different research perspectives and different areas have different problems of vulnerability [11,12]. Vulnerability is commonly defined by the Third Assessment Report of the International Panel on Climate Change (IPCC) as the degree to which a system is susceptible to the adverse effects of climate variability or extremes. Næss et al. [13] considered vulnerability as “A function of exposure, sensitivity, and adaptive capacity, generated by multiple factors and processes”. The vulnerability also indicates the extent to damage of the assets exposed to the forces generated by the hazard [14]. Researchers have a more comprehensive understanding of the concept of vulnerability. There are a variety of vulnerability assessment methods that are different in their methodologies, vulnerability frameworks, and index selections [15]. According to earlier works, vulnerability assessment methods can be categorized in three categories. The first is modeling methods that can evaluate depth, elevation, and the velocity of floods by using the frequency, magnitude, and shape of the hydrograph [16,17]. This approach requires a lot of data, is ineffective in the absence of data and, when the model lacks the necessary data, this can lead to a loss of decision effectiveness, confusing decision makers [11]. The second is implementing a vulnerability assessment based solely on the available historical disaster records or measurement data. This method is constructed on data collection from real flood hazards and their usage. This method is a simple approach but can be slightly inaccurate. Owing to the rapid development of urbanization, historical disaster data cannot effectively reflect the current urban situation [18]. The last is the vulnerability indicators method, which has been adapted to use the available data to provide a logical image of the vulnerability of a location. Vulnerability from floods has many dimensions, such as social, physical, economic, and political, which influence how floods affect inhabitants in varying ways and with different intensities. Many frameworks for assessing flood vulnerability exist. For instance, Cutter et al. [19] used a principle component analysis to aggregate county level socio-economic data to assess the social vulnerability of different municipalities in the US. The analytical hierarchy process (AHP) was used to identify risk elements and assign decision parameter weights for creating a flood vulnerability distribution map [20,21]. Kablan et al. [22] used the method for the improvement of vulnerability in Europe (MOVE) framework that integrates various indicators including environmental and societal aspects. Van et al. [23] calculated flood vulnerability indexes that considered social, economic, environmental, and physical indicators to assess flood vulnerability. Kashyap et al. [24] have assessed vulnerability to flooding combined characteristics relating to exposure, sensitivity, and adaptive capacity.

The selection of indicators has been widely discussed by researchers. Various flood indexes have been used to assess flood vulnerability. The indicators related to physical aspects, such as blocks with paved roads, health centers, sea-level rise [25,26,27], and increasing groundwater levels [28], are applicable to physical flood vulnerability and these parameters have been widely used. Physical, social, and infrastructure indicators have been widely used in national or regional flood vulnerability assessments. Chang et al. [29] selected indicators from social, ecological, and technological dimensions to assess urban flood vulnerability. However, with the acceleration of urbanization processes, populations and economies are highly concentrated in urban areas, adding to the uncertainties in the vulnerability analysis process [30]. Moreover, the disaster-bearing capacities of neighborhoods are interdependent and, because of their similar service conditions and structural behaviors, some indicators used in existing studies are not suitable for assessing flood vulnerability. The geographic information system (GIS) has been frequently adopted to evaluate the spatial heterogeneity of vulnerability [31]. Geographic information technology can be more widely used in index extraction. The aim of the existing studies is to be policy-making tools for reducing flood risk. Duan et al. [32] used game theory to determine the spatiotemporal distribution of the flood vulnerability. Yang et al. [10] developed a multiple flood vulnerability assessment approach based on the fuzzy comprehensive evaluation method (FCEM) and coordinated development degree model (CDDM) to reveal the internal relationships of exposure, sensitivity, and adaptive capacity. Helderop et al. [33] explored the interrelationships between social, geomorphic, and climatic factors, highlighting the many ways in which they contribute to somewhat unexpected vulnerabilities for coastal settlements. Urban flood vulnerability can be mitigated by effective land use and efficient urban drainage system [34]. Within the community, the legalization of certain materials and techniques for construction are effective to reduce flood vulnerability [35]. The proper planning of grey infrastructure and green infrastructure are important ways to reduce flood vulnerability [36]. The risk perception of the local population can also affect the urban flood risk [37]. However, in these studies, few researchers considered the relationship between indicators for assessing vulnerability. They have only focused on the vulnerability score. This does not intuitively provide policy recommendations for mitigating vulnerability.

The aim of this study is to provide a theoretical basis for the government to manage floods. For this aim, a framework was developed that integrates the physical, economic, community, nature, population, and political indicators. In particular, ArcGIS and the environment for visualizing images (ENVI) were used to extract the impermeable areas, fractional vegetation coverage, and built-up areas that have geographic attributes. Then, a contribution analysis of 18 individual indicators was performed. The process analysis method was applied to explore the drive mechanism pathways between the indicators. Based on these analyses, specific advice is then proposed for the study area to decrease the UFV.

### 1.2. Study Area

**Figure 1:**
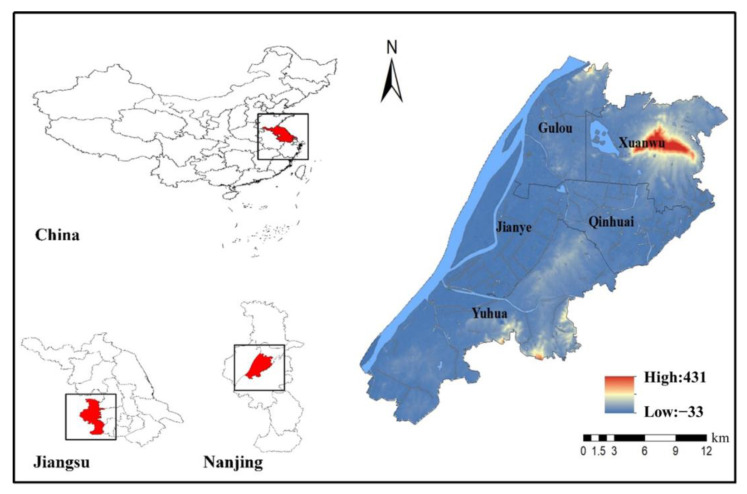
Administrative divisions of the research area.

The districts of Gulou, Jianye, Qinhuai, Xuanwu, and Yuhuatai are the core region of Nanjing City and they have high population densities and frequent human activities [38]. The region is located between latitude 31°14′ N–32°37′ N and longitude 118°22′ E–119°14′ E (Figure 1). The area is located in the north subtropical monsoon climate, with an annual precipitation of 1200 mm [39,40]. The average annual temperature is 15.4 °C, the highest annual temperature is 39.7 °C, and the lowest temperature is −13.1 °C. This region covers an area of 392.01 km^2^, accounting for 5.95% of Nanjing, and the population of this region is 3.7 million, accounting for 43% of Nanjing. Moreover, its gross domestic product (GDP) reached USD 58.68 billion in 2018. Geographically, this region lies on flat land with an elevation generally between 10- and 20 m above the sea level.

This region has a subtropical monsoon climate with distinct seasons and abundant rainfall. Under the control of the monsoonal climate, the winters are dry and the summers are rainy. According to the data statistics, the average annual rainfall days in the region is 117 days, the average rainfall amount is 1106.5 mm, and the relative humidity is 76%. In June every year, the region is prone to typhoons and frequent rainstorms, which can easily lead to disasters within the city. In recent years, floods have occurred almost every year in Nanjing, leading to the flooding of residential areas with imperfect drainage network construction and the paralysis of urban road traffic systems. In some years, this has occurred to a large degree and has had a wide coverage. The rapid development of urbanization has brought about a high concentration of population in the cities, causing cities to be vulnerable to floods. In addition, rapid urbanization has led to rapid growth in impermeable areas of the region, which contributes to the spatial and temporal redistribution of surface runoff, resulting in the greater magnitude and less concentration time of flood peaks in urbanized areas and creating conditions for urban floods [41].

## 2. Data and Methodology

### 2.1. The Urban Flood Vulnerability Model

**Figure 2:**
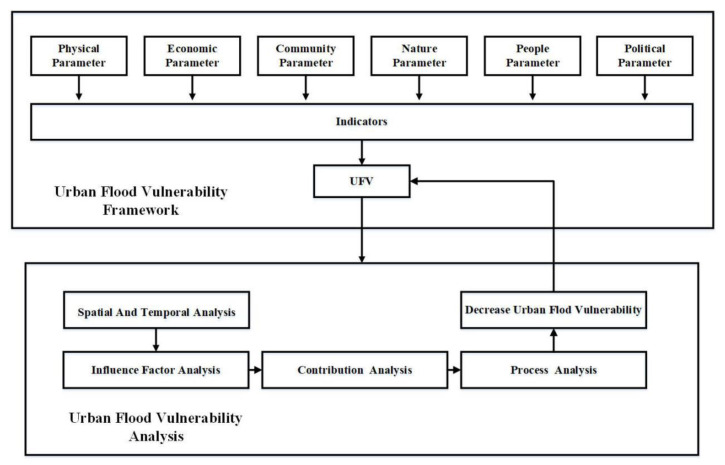
Overall study scheme.

The methodology used in this study agrees well with the goal of the study, which is the assessment of the vulnerability to urban flooding of the five districts of Nanjing. The study framework is shown in Figure 2. For the comprehensive consideration of the concept of vulnerability, 18 indicators were developed from six categories of physical, economic, community, nature, population, and political parameters, as obtained from the relevant literature regarding flood vulnerability and climate change vulnerability [10,42,43]. The explanation and references of the evaluation index system are shown in Table 1.

### 2.2. Calculation of the Urban Flood Vulnerability

#### 2.2.1. Standardized Flood Indexes

To calculate the final vulnerability score, the indictor set was first standardized using the Min-Max method in order to ensure the data are comparable and eliminate the effect of dimension. However, because standardization follows certain rules, it was important to assess the functional relationship between the indicator and the vulnerability. Thus, the positive and negative indexes were standardized using the following equations:

(1) $$x_{\mathrm{ij}}=\frac{x_{j}{-x}_{\min}}{x_{\max}{-x}_{\min}},$$

where $x_{\mathrm{ij}}$ = the standardized value; $x_{j}$ = the value of the indicator j; $x_{\max}$ = the maximum value of the indictor j; and $x_{\min}$ = the minimum value of the indicator j.

#### 2.2.2. Weighting Procedure

The weight of the indicators can be divided into objective and subjective weighting. The subjective weighting depends on the subjective factors and the amount of qualitative data was larger than that of quantitative data. The entropy method is an objective weighting method that can reflect the utility value of the index. Its weight value has higher credibility and accuracy than that of the subjective weighting method. Therefore, the entropy method was used in this study to calculate the weight index. The proportion of the year index value in comparison with all the indexes was calculated using the following equation:

(2) $$y_{\mathrm{ij}}=\frac{x_{\mathrm{ij}}}{\sum_{i=1}^{m}x_{\mathrm{ij}}}(0\leq y_{\mathrm{ij}}\leq 1),$$

where $y_{\mathrm{ij}}$ = the proportion of the index value of year i in the index of item j; and m = the total number of years. The entropy value was calculated using the following equation:

(3) $$e_{j}=-K\overset{m}{\underset{i=1}{\sum }}y_{\mathrm{ij}}{\mathrm{lny}}_{\mathrm{ij}}\;(K=\frac{1}{\mathrm{lnm}}),$$

where $e_{j}$ = the information entropy value of the index j.

The weighting was performed using the following equation:

(4) $$w_{j}=\frac{d_{j}}{\sum_{i=1}^{m}d_{j}},$$

where $d_{j}\;$= the information utility value of the index j; and $w_{j}$ = the weighting of the index j.

#### 2.2.3. Urban Flood Vulnerability Score

The vulnerability score was calculated using the following equation:

(5) $$\mathrm{UFV}=\overset{n}{\underset{i=1}{\sum }}y_{\mathrm{ij}}w_{j}.$$

### 2.3. Impermeable Area

**Figure 3:**
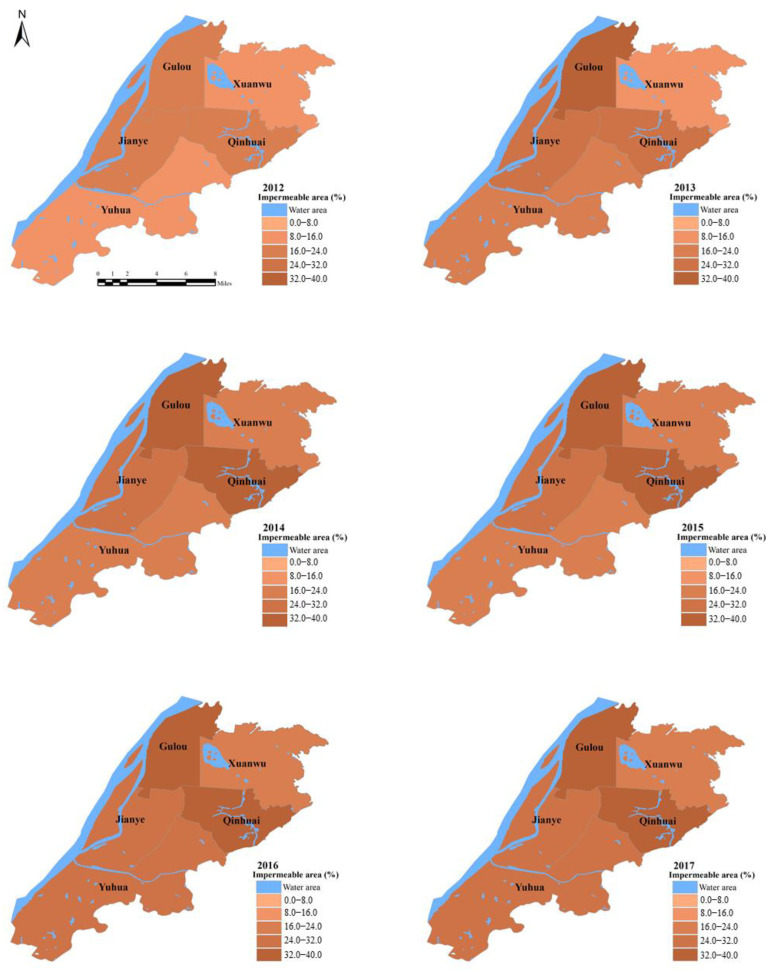

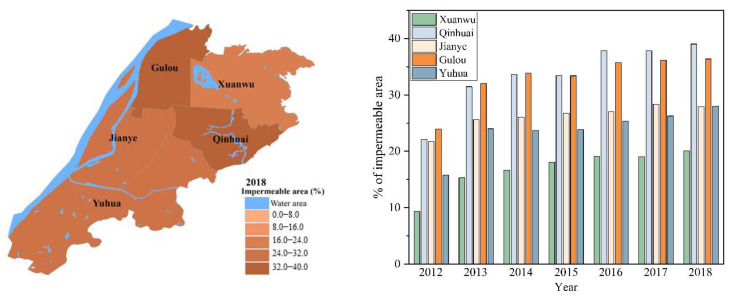
Spatial distribution maps of the impermeable area from 2012 to 2018 for the five districts.

The impervious surface refers to the artificial surface. The biggest feature of this type of surface is that it can prevent surface water from infiltrating into the soil. A spectral blending analysis is a common method for spectral blending decomposition. It is used to calculate the composition proportion of different end elements in mixed pixels and decompose the spectrum of mixed pixels into a combination of spectral information of various end elements and ground types. Spectral hybrid decomposition models can be divided into linear and nonlinear models according to the relationship between variables. However, many parameters in the nonlinear spectral hybrid decomposition model are difficult to measure accurately and obtain. The linear spectral mixed decomposition model was used in this study to assess the impermeable area based on the ENVI. The linear spectral mixed decomposition model is one of the most commonly used methods to obtain the impermeable surface information of the medium spatial resolution percentage of subpixels. The principle is to assume that the reflectance of a pixel in a certain spectral band is a linear combination of the reflectance of the basic components forming the pixel and the proportion of the area of the pixel as the weight coefficient. The specific steps are as follows. First, it is necessary to preprocess the image, including the geometric correction, atmospheric correction, and image cutting. Second, the minimum noise fraction (MNF) is calculated due to remote sensing image spectral correlation. The occurrence of noise reduces the amount of information remote sensing data images required to be an effective analysis method. Therefore, researchers need to reduce the spectral correlation between the separation and noise of remote sensing data at the same time. Using a superimposed MNF transform principal component transformation can effectively produce multispectral and hyperspectral data dimension reduction more effectively than a principal component analysis to reduce the data dimension, isolate information noise, and reduce the correlation between bands, which reduces the computational burden. Third is the selection of end elements. According to the end element selection process, the end element is divided into a reference end element and an image end element. Image end elements are selected from the image to be classified and are modified and adjusted continuously and finally determined. All the image end elements are considered as a linear combination of image end elements. The reference is the spectrum from the spectrum library, so the use of a reference to conduct the decomposition of mixed pixels and the CNY has a higher accuracy in theory. However, the video imaging can be affected by the sensor and uncertain factors such as the atmosphere. Therefore, the features of the spectral curve and field measurements and the feature spectrum curve of the unbridled differences, even after a series of pretreatments, are also difficult to perfectly determine using the spectral feature spectrum curve in the repository. The end elements obtained from the image have the same measurement scale as the image data. In addition, due to the lack of a mature spectrum library suitable for a specific area, field measurements often require a lot of labor and material resources and this has become the primary means to obtain the end elements from the image itself. Owing to the complex composition of ground objects on the urban surface and the large effect of the urban atmosphere on remote sensing images, studies have shown that image end elements can be directly extracted from images and mixed pixel decomposition has a better effect. Therefore, the end elements used in this study were directly extracted from images. Fourth, the selection of the end member and the selection of the end elements determine the precision of the result of the mixed pixel decomposition. The number of end elements and the fitting degree of the decomposition model need a balance; too many end elements will cause the decomposition model to be too sensitive to the end element selection. If the end element is too small, the suitability of the model will be insufficient to fully explain the spectral changes. For the extraction and analysis of urban impermeable ground, it is more ideal to select four end elements, that is, four end elements with high reverse illumination, low reverse illumination, vegetation, and soil, for the decomposition of mixed pixels. Fifth, the building of the water mask. Because a water body is a ground object with low reverse illumination, the influence of a water body must be eliminated before calculating the impermeable surface coverage; a water body mask should be applied to the image. After the mask, the value region is processed and the shielded value region is excluded from the calculated range. Finally, the high albedo image and ground albedo image after the linear spectral mixed decomposition are added and the impermeable layer is obtained. The data were obtained from the U.S. geological survey network (https://ers.cr.usgs.gov/login (accessed on 5 November 2021)). Additionally, the percentage of impermeable area in the five districts are shown in Figure 3. The percent of impermeable area of all districts showed a continuous rising trend. Qinhuai and Gulou have the highest percent of impermeable area from 2012 to 2018.

### 2.4. Fractional Vegetation Cover

The fractional vegetation cover (FVC) refers to the percentage of the vertical projection area of vegetation (including leaves, stems, and branches) on the ground to the total area of the statistical area. In this study, the FVC was calculated by the dimidiate pixel model using the ENVI. The dimidiate pixel model is a simple and practical remote sensing estimation model; it assumes the pixel surface by using the vegetation-covered portions of the surface and surface portions of the vegetation coverage. Then, the spectral information from the remote sensing sensor observed, using these two components factored into the linear weighted synthesis, that the weight of each factor is their respective areas as a ratio in pixels, as the vegetation coverage can be thought of as the weight of vegetation. Based on the dimidiate pixel model and the normalized difference vegetation index, the vegetation fraction of the study region can be calculated by using the model for quantifying the vegetation fraction from the normalized difference vegetation. The FVC was performed using the following equation:

(6) FVC=(NDVI − NDVImin)/(NDVImax − NDVImin).

### 2.5. Built-Up Area

The built-up area was obtained from the MODIS land cover data using GIS. The MODIS land cover data describes land cover types based on one-year of Terra and Aqua observations. The land cover dataset consists of 17 primary land cover types, including 11 natural vegetation types, 3 developed and embedded land types, and 3 non-vegetative land type definition classes according to the International Geosphere Biosphere Programme (IGBP). MCD12Q1, the annual global 500 m product of MODIS Terra + Aqua, adopts five different land cover classification schemes and the primary technology for information extraction is to supervise the classification of decision trees.

## 3. Results

Recognizing the spatiotemporal characteristics of the UFV is the basis for reducing vulnerability and risk. A contribution analysis contributed to this exploration of UFV reduction.

### 3.1. Spatial and Temporal Analysis

**Figure 4:**
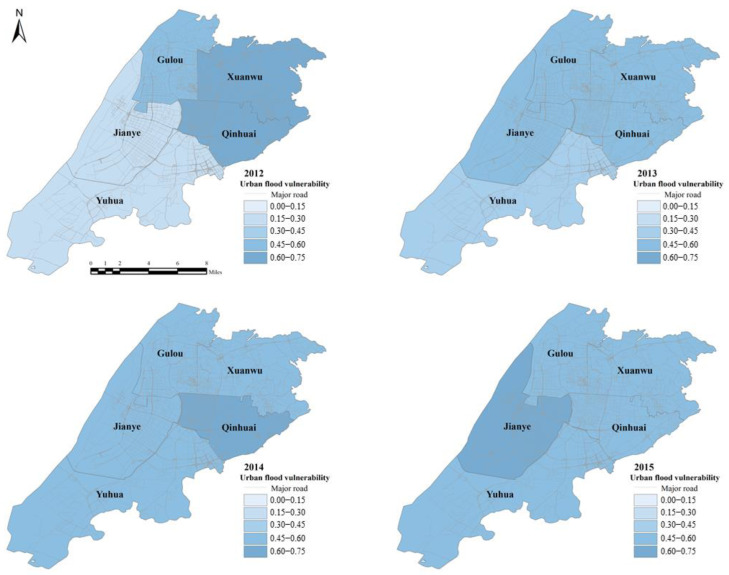

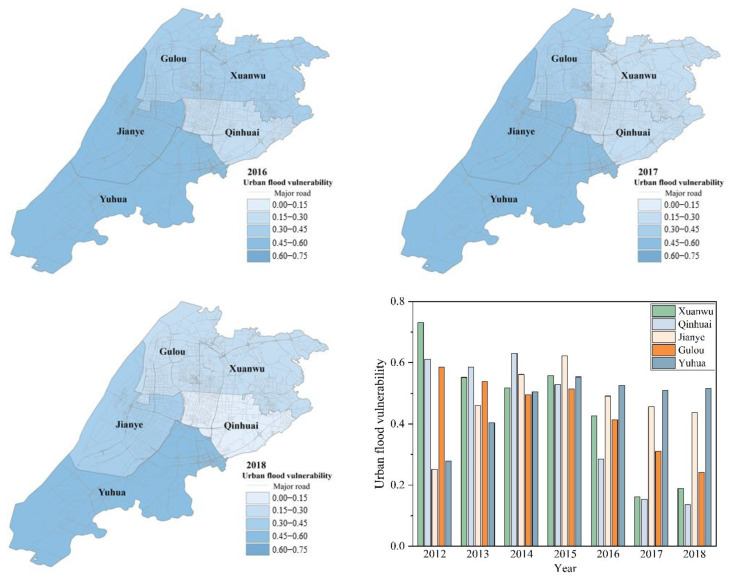
The UFV spatial distribution maps of the XQJGY region.

The trends of five districts from 2012 to 2018 are shown in Figure 4. For Jianye and Yuhua, the trend of vulnerability rose and reached the maximum value in 2015, then it declined. The amplitude of the fluctuations had a larger trend than that in the other districts. In Xuanwu, the UFV decreased from the highest point from 2012 to 2014, increased from 2014 to 2015, then decreased to the lowest point from 2015 to 2017, and it increased from 17 to 18 years. Except for an increase in 2013, Qinhuai showed a downward trend every year. Similarity, Gulou presented an upward trend in 2014. Yuhua showed a steady increase from 2012 to 2015 and in the following years it remained comparatively stable. Jianye jumped up from 0.26 in 2012 to 0.61 in 2015, then fell back to 0.44 in 2018.

The spatial distribution maps of the UFV of the five districts are shown in Figure 4. The UFV of these five districts is spatially heterogeneous. In 2012 and 2013, Xuanwu and Qinhuai have the highest UFV. The UFV of Qinhuai and Jianye is higher than other districts in 2014. The UFV in Jianye continued to increase from 2012 to 2015 and the urban flood vulnerability in 2015 was significantly higher than that in the other districts. The UFV of Yuhua remained stable and was the most vulnerable from 2016 to 2018, while it showed a downward trend in other districts. Qinhuai had the lowest UFV in 2018. Jianye and Yuhua has the lowest vulnerability of urban flood in 2012, but the UFV of the two districts increased to become the highest in 2018. The UFV of Gulou is at a medium or low level in the five districts from 2012 to 2018.

### 3.2. Contribution Analysis

#### Contribution Analysis of the Five Indicators

**Figure 5:**
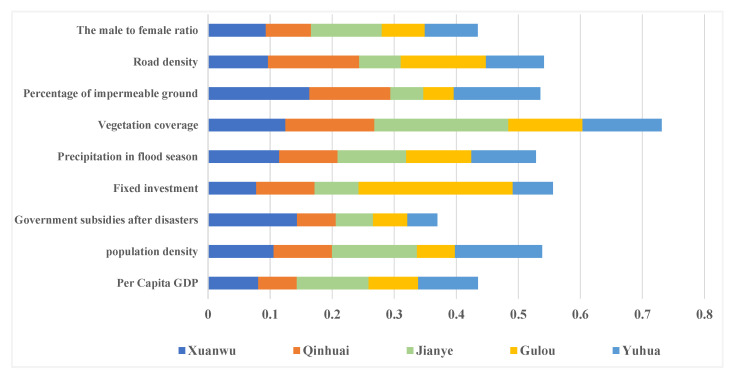
The contribution of indicators to urban flood.

The contributions of the indicators to the UFV for each district are illustrated in Figure 5. Because there are 21 indicators, this study analyzed the top 6 indicators of the five districts for the index contribution analysis. The top six indicators for each district are similar but differ in their detail. For all the districts, the vegetation coverage, precipitation during the flood season, population density, and road density are in the top six contributors. In the Xuanwu district, the impermeable area contributed the most to the UFV. Similarly, the third largest contributor in the Qinhuai districts was the impermeable area.

(1) Vegetation Coverage

For all the districts, the proportion of vegetation coverage to the UFV was greater than 10%. The contribution of vegetation coverage in the Jianye district was the largest, at 21.6%. The vegetation coverage of the Gulou district contributed the smallest amount to the UFV (11.96%).

(2) Precipitation during the Flood Season

Heavy rainfall can cause urban flooding. According to historical disaster data, it was stipulated in the 2015 edition of the Nanjing Flood Control Planning that Nanjing City had its annual flood season from May to September. During this period, Nanjing is more vulnerable to flood disasters. Extreme precipitation events are more likely to occur and rainstorm floods are more likely to occur in local vulnerable areas. It can be seen from the contribution index that in the five districts, the precipitation during the flood season was one of the first five indicators. Xuanwu had the largest share (11.45%), followed by Jianye (11.06%), Gulou (10.51%), and Yuhua (10.42%).

(3) Impermeable Surface Area

The districts where the impermeable surface area was one of the dominant factors of the UFV were Xuanwu and Qinhuai. Xuanwu accounted for 16.33% and Qinhuai accounted for 13.06%. It is understandable that the less impermeable surface area in a city will accelerate the speed of rainwater infiltration and reduce the possibility of rainwater accumulation, thus reducing the possibility of urban flooding.

### 3.3. The Mediating Effect Test

**Figure 6:**
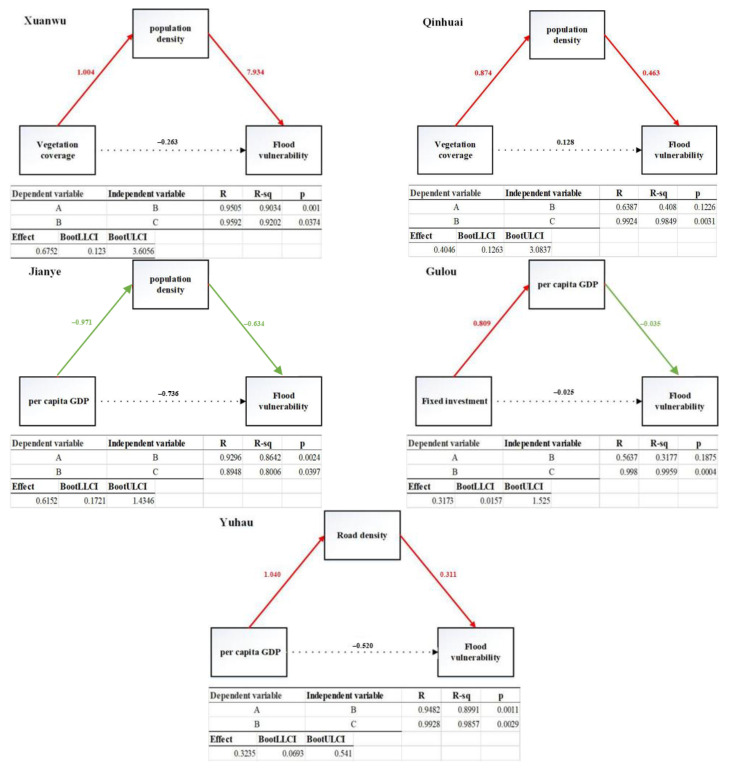
The path models of the five districts.

**Figure 7:**
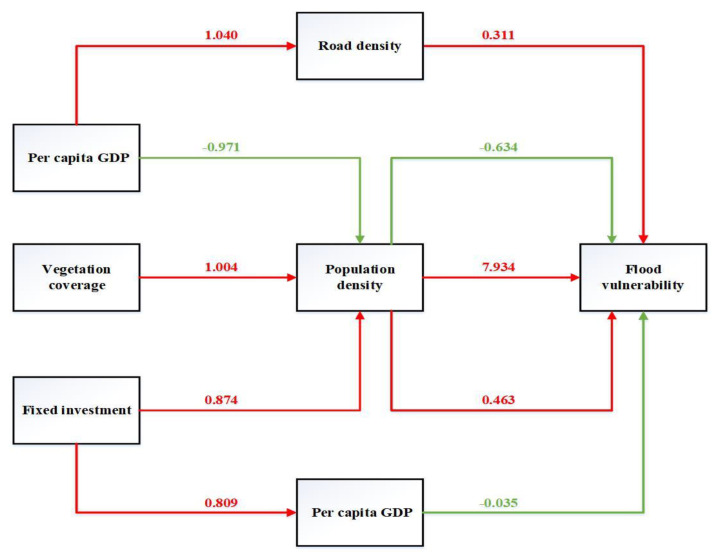
The integrated path models.

The mediating effect refers to how the effect of A on C is realized by B, that B is a function of A, and C is a function of B (A-B-C) [44]. Considering the influence of an independent variable A on a dependent variable C, if A influences variable C through B, then B is called the intermediary variable. In recent years, the bootstrap method has been utilized in most studies published in top international academic journals in psychology, consumer behavior, organizational behavior, and other fields to test the mediating effect. This study also used this method to test whether there is a mediating effect between indicators. Mediation path models (Figure 6) and an integrated path diagram for each district were then established (Figure 7). The solid line is the indirect effect path and the dotted line indicates the direct influence path. The red line shows positive effects, while the green line shows negative effects.

#### 3.3.1. Xuanwu District

The mediating effect of “vegetation coverage-population density-flood vulnerability” was significant, while the direct effect of “vegetation coverage-flood vulnerability” was not significant. In this intermediary path, vegetation coverage reflects the greening environment of the region. The greening environment has a significant effect on the agglomeration effect of the regional population. There are many residential communities around the green space of the park and the population density is large. Therefore, the vegetation coverage was positively correlated with the population density. The greater the population density, the higher the vulnerability of the region to flood disaster, so the population density was positively correlated with the flood vulnerability.

#### 3.3.2. Qinhuai District

The mediating effect of “fixed investment-population density-flood vulnerability” was significant, while the direct effect of “fixed investment-flood vulnerability” was not significant. In the intermediary path, the higher the fixed investment was, the better the regional government’s financial situation was, and there was enough fiscal revenue to support the higher fixed investment. The per capita GDP reflects the overall economic level of the region; hence, fixed investment was positively correlated with per capita GDP. The greater the population density, the higher the vulnerability of the region to flood disaster. Therefore, the population density was positively correlated with the flood vulnerability.

#### 3.3.3. Yuhua District

The mediating effect of “per capita GDP-road density-flood vulnerability” was significant, while the direct effect of “per capita-flood vulnerability” was significant. In the mediation path, the per capita GDP reflects the economics of area residents. The economic level is higher in urban areas where urbanization levels are high and these areas will have a higher road density. Therefore, there was a positive correlation between the per capita GDP and road density and road density; the higher the representative regional transportation routes, which receive urban flooding operation losses, the greater the increase in the flood vulnerability of the area. Therefore, the road density had a negative correlation with the flood vulnerability. In the direct path, the per capita GDP reflects the regional economic level. The higher the economic level is, the stronger the resilience, risk resistance, and post-disaster recovery will be in the face of floods and flood disasters. Therefore, the per capita GDP was negatively correlated with flood vulnerability.

#### 3.3.4. Jianye District

The mediating effect of “per capita GDP-population density-flood vulnerability” was significant and the direct effect of “per capita-flood vulnerability” was also significant. The per capita GDP was negatively correlated with the population density. A higher per capita GDP means better personal economic levels and residents with better economic levels are able to live in high-end residential areas, which have smaller plot ratios and lower population densities.

#### 3.3.5. Gulou District

The mediating effect of “fixed investment-per capita GDP-flood vulnerability” was significant and the direct effect of “fixed investment-flood vulnerability” was significant. In the intermediary path, the higher the fixed investment was, the better the regional government’s financial situation was, and there was enough fiscal revenue to support the higher fixed investment. The per capita GDP reflects the overall economic level of the region and the fixed investment was positively correlated with the per capita GDP. The higher the economic level, the stronger the resilience, risk resistance, and post-disaster recovery in the face of floods and flooding disasters. Therefore, there was a negative correlation between the per capita GDP and vulnerability. In the direct path, fixed investment was negatively correlated with vulnerability. The higher the fixed investment was, the more funds the regional government has. Investment in infrastructure will increase the stability of infrastructure. This can effectively reduce the flood disaster vulnerability.

#### 3.3.6. By Combining the Paths of the Five Regions, the Comprehensive Path Was Plotted

The per capita GDP, road density, and flood vulnerability had a positive conversion path. The per capita GDP, population density, and flood vulnerability had a negative conversion path. The vegetation coverage, population density, and flood vulnerability had a positive conversion path. The fixed investment, population density, and flood vulnerability had a positive conversion path. The fixed investment, per capita GDP, and flood vulnerability had a positive conversion path.

## 4. Discussion

Based on the index-based system method, this study assessed the UFV of the XQJGY region in Nanjing and performed the contribution analysis of different indicators. The XQJGY region showed the heterogeneity in the temporal and spatial distribution of UFV. Flood vulnerability represented the decreasing trend in almost all the districts from 2012 to 2018, except the Yuhua and Jianye districts. Additionally, many researchers have found the analogous results of urban flood characteristics by using Nanjing as a case study, which was believed to have flood resilience [45] and lower the exposure risk [46]. UFV presented the aggregation effect in the spatial distribution and was primarily concentrated on the southern XQJGY region, which was consistent with the current studies [47,48]. This study also emphasized and extracted the indicators with geographic attributes and created the contribution analysis in UFV. Additionally, vegetation coverage showed the highest contribution to flood vulnerability in all the districts and the impermeable surface area contributed the most in the Xuanwu and Qinhuai districts. Rapid urbanization dramatically changed the feature of the urban underling surface [49] and the natural surface, such as farmland and woodland, was sharply decreased and obviously converted into the impermeable surface [50]. Unbalanced land transformation significantly affected the urban hydrological system [51] and prevented rainwater from infiltrating into the ground [52], which would intensify the urban flood risk faced with climate change. Although flood vulnerability showed the decreasing trend in the XQJGY region, it was also necessary to pay attention to the change of vegetation cover and impervious surface and avoid the large-scale urbanization for a more resilient city.

This study explored the influence path of flood vulnerability and revealed the mediating effect between indicators in different districts. It was found that the road density showed a positive effect on flood vulnerability, which was in agreement with the results of the present study [53]. The road density was the common significant factor affecting the urban flood and negatively correlated with floods in Chinese megacities [46,53]. The most-connected road links within a network were more prone to floods and urban floods became more frequent with the increase in road density [54]. The roads were believed to disrupt water storage and affect the infiltrated volume and consequently increase flood vulnerability [55]. We found that the per capita GDP showed a negative effect on flood vulnerability. The per capita GDP mainly reflected the per capita economic development level and performance [45] in the XQJGY region. The functioning economy system could support the capacity to resist to, adapt to, and recover from flood events in cities [56,57]. Additionally, the vitality, redundancy, and resourcefulness of the local economy helped to enhance individuals’ adaptive capacity of flood prevention and the economic affluence caused people to be more resilient to floods [56], which could mitigate and reduce flood vulnerability. Furthermore, vegetation coverage showed the indirect negative effect on flood vulnerability and population density played the mediating effect between vegetation coverage and flood vulnerability. In current studies, good vegetation coverage, positively correlated with floods, was believed to provide the ecosystem service of flood mitigation [58,59], reduce the flood peak and regulate the hydrological process [60] through precipitation interception and soil infiltration [61]. However, vegetation coverage could increase population density and lead to flood vulnerability according to this study. Vegetation promoted human behavior and personal perception and showed the benefits of microclimate mitigation and thermal comfort improvement at the neighborhood scale [62]. However, increased anthropogenic activities could enhance the probability of flood occurrence [53] and aggravate flood risk exposure [45] and population density was demonstrated to have the significant positive correlation with flooding [46,47]. Therefore, it was fundamental to attach importance to the rational planning and recovery of vegetation for inhibiting the positive effect of vegetation coverage on flood vulnerability.

The physical expansion of urban areas often arose at the expense of natural space [63] and rapid urban growth posed pressure on sustainable development and led to a high flood risk. Additionally, the increased impervious areas ought to adopt responsibility for more frequent flood events. The most developing countries mainly depended on grey solutions to achieve flood management, such as dams or concrete pipes, but this approach lacks sufficient sustainability and resilience [64]. Based on nature-based solutions, blue-green infrastructure (BGI) referred to the interconnected infrastructure network of natural or man-made green and blue spaces [65], including forests, wetlands, green roofs, green walls, rivers, creeks, parks, etc. BGI promoted ecosystem resilience and human well-being by providing a range of ecosystem services [66] and operated as the useful and effective way to mitigate and adapt to floods. Therefore, based on the results of this study, we recommended these suggestions to promote the development of BGI in the XQJGY region for mitigating flood vulnerability and increasing flood resilience. First, reasonably control the urbanization process to avoid excessive urban expansion in order to reduce the increase in or generation of impervious surfaces. Additionally, pay attention to the protection of urban vegetation coverage and rationally plan the vegetation distribution to enhance the flood regulation effect of the urban landscape. Furthermore, build and restore urban rivers, lakes, water systems, green spaces, and parks and improve the capacity of blue-green spaces to absorb rainwater. Finally, effectively protect and fully utilize urban blue and green infrastructure and promote the coordination role of blue, green, and gray systems.

This study stressed the role of indicators with geographic attributes in flood vulnerability and illustrated the influence path and mediating effect. Although these paths provided the theoretical basis for mitigating flood vulnerability, how to put into practice remained challengeable and unsolved. Current studies focused on flood simulation and modeling [50] and attempted to reveal the hydrologic process with climate change [67,68]. Additionally, flood simulation could be used to examine and test the actual influence of vulnerability indicators through the change of modeling parameters. Moreover, although major rainstorm occurring on the large impervious areas became the primary source and cause of urban flooding [69], rising river levels and flash floods were also a contribution factor for floods [67,70] and led to the considerable losses during heavy rain. Future studies should further explore the cascading effect and influence the consequence between different flooding types.

## 5. Conclusions

In this article, a method for measuring and mapping flood vulnerability was demonstrated using Xuanwu, Qinhuai, Jianye, Gulou, and Yuhua as examples. The results contribute to a growing body in the literature on flood vulnerability assessments, which offer some perspectives for both researchers and policy makers. One of the key perspectives of this study was that an integrative framework was used for measuring the flood vulnerability that included physical, economic, community, nature, population, and policy parameters. The flood vulnerability of the affected study area utilized a composite index and was based on geographic information technology. Another perspective was the use of a contribution analysis and process analysis from the index layer, which was conducive for the exploration of the influencing factors and transformation path of the UFV.

The findings demonstrated that the flood vulnerabilities in the XQJGY region were different. From 2012 to 2018, the UFV of the Yuhua district and Jianye district increased, while Xuanwu, Qinhuai, and Gulou decreased. By using an analysis of the specific contribution of the index layer, it was observed that the top six indicators that significantly contributed to the UFV were the following: vegetation coverage, precipitation during the flood season, population density, and road density.

This study analyzed the UFV of the XQJGY region and its influential factors. Based on the results, the interaction mechanisms of the cities in the XQJGY region should be explored in future studies.

## Figures and Tables

**Table 1:** Indicators of urban flood vulnerability.

Indicator	Category	Source
Per capita GDP (CNY)	Economic	2012–2018 statistical yearbook of Nanjing, 2012–2018 Xuanwu almanac, 2012–2018 Jianye almanac, 2012–2018 Yuhua almanac, 2012–2018 Gulou almanac, 2012–2018 Qinhuai almanac, 2012–2013 Xiaguan almanac, 2012–2013 Baixia almanac.
Proportion of male and female (%)	People	
Population density (person/km^2^)	People	
Government subsidies after disasters (10^4^ CNY)	Political	
Number of firefighters	Political	
Number of community hospitals	Political	
Fixed investment (10^8^/CNY)	Political	
Neighborhood committee coverage ability	Community	
Number of registered volunteers	Community	
Precipitation in flood season (mm)	Nature	
Vegetation coverage	Nature	
Number of old plots	Physical	
Number of protected buildings	Physical	
The percentage of impermeable ground (%)	Physical	
Built-up area (square meter)	Physical	
Road density (km/km^2^)	Physical	
Water area (km^2^)	Physical	
Length of vulnerable road (km)	Physical	

## Data Availability

All data included in the analysis during this study are available on request from the corresponding author.

## References

[1] Asadi Zarch M.A., Sivakumar B., Sharma A. (2015). Droughts in a Warming Climate: A Global Assessment of Standardized Precipitation Index (SPI) and Reconnaissance Drought Index (RDI). J. Hydrol..

[2] Balaguru K., Foltz G.R., Leung L.R., Emanuel K.A. (2016). Global Warming-Induced Upper-Ocean Freshening and the Intensification of Super Typhoons. Nat. Commun..

[3] Arnell N.W., Gosling S.N. (2016). The Impacts of Climate Change on River Flood Risk at the Global Scale. Clim. Chang..

[4] Cannaby H., Fach B.A., Arkin S.S., Salihoglu B. (2015). Climatic Controls on Biophysical Interactions in the Black Sea under Present Day Conditions and a Potential Future (A1B) Climate Scenario. J. Mar. Syst..

[5] Bell S. (2011). Water and the City: Risk, Resilience and Planning for a Sustainable Future. Int. J. Urban Sustain. Dev..

[6] Sun Y., Chau P.H., Wong M., Woo J. (2017). Place- and Age-Responsive Disaster Risk Reduction for Hong Kong: Collaborative Place Audit and Social Vulnerability Index for Elders. Int. J. Disaster Risk Sci..

[7] Doocy S., Daniels A., Murray S., Kirsch T.D. (2013). The Human Impact of Floods: A Historical Review of Events 1980-2009 and Systematic Literature Review. PLoS Curr..

[8] Mustafa A., Bruwier M., Archambeau P., Erpicum S., Pirotton M., Dewals B., Teller J. (2018). Effects of Spatial Planning on Future Flood Risks in Urban Environments. J. Environ. Manag..

[9] Du S., He C., Huang Q., Shi P. (2018). How Did the Urban Land in Floodplains Distribute and Expand in China from 1992–2015?. Environ. Res. Lett..

[10] Yang W., Xu K., Lian J., Bin L., Ma C. (2018). Multiple Flood Vulnerability Assessment Approach Based on Fuzzy Comprehensive Evaluation Method and Coordinated Development Degree Model. J. Environ. Manag..

[11] Balica S.F., Popescu I., Beevers L., Wright N.G. (2013). Parametric and Physically Based Modelling Techniques for Flood Risk and Vulnerability Assessment: A Comparison. Environ. Model. Softw..

[12] Nasution B.I., Saputra F.M., Kurniawan R., Ridwan A.N., Fudholi A., Sumargo B. (2022). Urban Vulnerability to Floods Investigation in Jakarta, Indonesia: A Hybrid Optimized Fuzzy Spatial Clustering and News Media Analysis Approach. Int. J. Disaster Risk Reduct..

[13] Næss L.O., Norland I.T., Lafferty W.M., Aall C. (2006). Data and Processes Linking Vulnerability Assessment to Adaptation Decision-Making on Climate Change in Norway. Glob. Environ. Chang..

[14] Bouaakkaz B., El Morjani Z.E.A., Bouchaou L. (2023). Social Vulnerability Assessment to Flood Hazard in Souss Basin, Morocco. J. African Earth Sci..

[15] Huang D., Zhang R., Huo Z., Mao F., E Y., Zheng W. (2012). An Assessment of Multidimensional Flood Vulnerability at the Provincial Scale in China Based on the DEA Method. Nat. Hazards.

[16] Ouyang M., Kotsuki S., Ito Y., Tokunaga T. (2022). Employment of Hydraulic Model and Social Media Data for Flood Hazard Assessment in an Urban City. J. Hydrol. Reg. Stud..

[17] Luo P., Luo M., Li F., Qi X., Huo A., Wang Z., He B., Takara K., Nover D., Wang Y. (2022). Urban Flood Numerical Simulation: Research, Methods and Future Perspectives. Environ. Model. Softw..

[18] Nasiri H., Mohd Yusof M.J., Mohammad Ali T.A. (2016). An Overview to Flood Vulnerability Assessment Methods. Sustain. Water Resour. Manag..

[19] Cutter S.L., Boruff B.J., Shirley W.L. (2003). Social Vulnerability to Environmental Hazards. Soc. Sci. Q..

[20] Palacio-Aponte A.G., Ortíz-Rodríguez A.J., Sandoval-Solis S. (2022). Methodological Framework for Territorial Planning of Urban Areas: Analysis of Socio-Economic Vulnerability and Risk Associated with Flash Flood Hazards. Appl. Geogr..

[21] Ouma Y.O., Tateishi R. (2014). Urban Flood Vulnerability and Risk Mapping Using Integrated Multi-Parametric AHP and GIS: Methodological Overview and Case Study Assessment. Water.

[22] Kablan M.K.A., Dongo K., Coulibaly M. (2017). Assessment of Social Vulnerability to Flood in Urban Côte d’Ivoire Using the MOVE Framework. Water.

[23] Van C.T., Tuan N.C., Son N.T., Tri D.Q., Anh L.N., Tran D.D. (2022). Flood Vulnerability Assessment and Mapping: A Case of Ben Hai-Thach Han River Basin in Vietnam. Int. J. Disaster Risk Reduct..

[24] Kashyap S., Mahanta R. (2021). Chapter 27-Socioeconomic Vulnerability to Urban Floods in Guwahati, Northeast India: An Indicator-Based Approach.

[25] Jonkman S.N., Hillen M.M., Nicholls R.J., Kanning W., Ledden M. (2013). van Costs of Adapting Coastal Defences to Sea-Level Rise—New Estimates and Their Implications. J. Coast. Res..

[26] Lasage R., Veldkamp T.I.E., de Moel H., Van T.C., Phi H.L., Vellinga P., Aerts J.C.J.H. (2014). Assessment of the Effectiveness of Flood Adaptation Strategies for HCMC. Nat. Hazards Earth Syst. Sci..

[27] Storch H., Downes N.K. (2011). A Scenario-Based Approach to Assess Ho Chi Minh City’s Urban Development Strategies against the Impact of Climate Change. Cities.

[28] Rotzoll K., Fletcher C.H. (2013). Assessment of Groundwater Inundation as a Consequence of Sea-Level Rise. Nat. Clim. Chang..

[29] Chang H., Pallathadka A., Sauer J., Grimm N.B., Zimmerman R., Cheng C., Iwaniec D.M., Kim Y., Lloyd R., McPhearson T. (2021). Assessment of Urban Flood Vulnerability Using the Social-Ecological-Technological Systems Framework in Six US Cities. Sustain. Cities Soc..

[30] Chen W., Wang X., Deng S., Liu C., Xie H., Zhu Y. (2019). Integrated Urban Flood Vulnerability Assessment Using Local Spatial Dependence-Based Probabilistic Approach. J. Hydrol..

[31] Peng J., Zhang J. (2022). Urban Flooding Risk Assessment Based on GIS- Game Theory Combination Weight: A Case Study of Zhengzhou City. Int. J. Disaster Risk Reduct..

[32] Duan Y., Xiong J., Cheng W., Wang N., He W., He Y., Liu J., Yang G., Wang J., Yang J. (2022). Assessment and Spatiotemporal Analysis of Global Flood Vulnerability in 2005–2020. Int. J. Disaster Risk Reduct..

[33] Helderop E., Grubesic T.H. (2019). Social, Geomorphic, and Climatic Factors Driving U.S. Coastal City Vulnerability to Storm Surge Flooding. Ocean Coast. Manag..

[34] Rana I.A., Asim M., Aslam A.B., Jamshed A. (2021). Disaster Management Cycle and Its Application for Flood Risk Reduction in Urban Areas of Pakistan. Urban Clim..

[35] Nofal O.M., van de Lindt J.W. (2020). High-Resolution Approach to Quantify the Impact of Building-Level Flood Risk Mitigation and Adaptation Measures on Flood Losses at the Community-Level. Int. J. Disaster Risk Reduct..

[36] Li L., Uyttenhove P., Van Eetvelde V. (2020). Planning Green Infrastructure to Mitigate Urban Surface Water Flooding Risk—A Methodology to Identify Priority Areas Applied in the City of Ghent. Landsc. Urban Plan..

[37] Chen J., Chen W., Huang G. (2021). Assessing Urban Pluvial Flood Resilience Based on a Novel Grid-Based Quantification Method That Considers Human Risk Perceptions. J. Hydrol..

[38] Chuai X., Feng J. (2019). High Resolution Carbon Emissions Simulation and Spatial Heterogeneity Analysis Based on Big Data in Nanjing City, China. Sci. Total Environ..

[39] Wang C., Sheng Y., Wang J., Wang Y., Wang P. (2022). Air Pollution and Human Health: Investigating the Moderating Effect of the Built Environment. Remote Sens..

[40] Wang P., Yu P., Huang L., Zhang Y. (2022). An Integrated Technical, Economic, and Environmental Framework for Evaluating the Rooftop Photovoltaic Potential of Old Residential Buildings. J. Environ. Manag..

[41] Liu J., Shi Z., Wang D. (2016). Measuring and Mapping the Flood Vulnerability Based on Land-Use Patterns: A Case Study of Beijing, China. Nat. Hazards.

[42] Sarkodie S.A., Strezov V. (2019). Economic, Social and Governance Adaptation Readiness for Mitigation of Climate Change Vulnerability: Evidence from 192 Countries. Sci. Total Environ..

[43] Tapia C., Abajo B., Feliu E., Mendizabal M., Martinez J.A., Fernández J.G., Laburu T., Lejarazu A. (2017). Profiling Urban Vulnerabilities to Climate Change: An Indicator-Based Vulnerability Assessment for European Cities. Ecol. Indic..

[44] Wang P., Yu P., Lu J., Zhang Y. (2022). The Mediation Effect of Land Surface Temperature in the Relationship between Land Use-Cover Change and Energy Consumption under Seasonal Variations. J. Clean. Prod..

[45] Wang P., Li Y., Zhang Y. (2021). An Urban System Perspective on Urban Flood Resilience Using SEM: Evidence from Nanjing City, China. Nat. Hazards.

[46] Li C., Liu M., Hu Y., Wang H., Zhou R., Wu W., Wang Y. (2022). Spatial Distribution Patterns and Potential Exposure Risks of Urban Floods in Chinese Megacities. J. Hydrol..

[47] Wang Y., Li C., Liu M., Cui Q., Wang H., LV J., Li B., Xiong Z., Hu Y. (2022). Spatial Characteristics and Driving Factors of Urban Flooding in Chinese Megacities. J. Hydrol..

[48] Wang B., Loo B.P.Y., Zhen F., Xi G. (2020). Urban Resilience from the Lens of Social Media Data: Responses to Urban Flooding in Nanjing, China. Cities.

[49] Luo K., Zhang X. (2022). Increasing Urban Flood Risk in China over Recent 40 Years Induced by LUCC. Landsc. Urban Plan..

[50] Wang P., Li Y., Yu P., Zhang Y. (2021). The Analysis of Urban Flood Risk Propagation Based on the Modified Susceptible Infected Recovered Model. J. Hydrol..

[51] Wang J., Liu J., Mei C., Wang H., Lu J. (2022). A Multi-Objective Optimization Model for Synergistic Effect Analysis of Integrated Green-Gray-Blue Drainage System in Urban Inundation Control. J. Hydrol..

[52] Liu Y., Huang X., Yang H. (2022). An Integrated Approach to Investigate the Coupling Coordination between Urbanization and Flood Disasters in China. J. Clean. Prod..

[53] Rahman M., Ningsheng C., Mahmud G.I., Islam M.M., Pourghasemi H.R., Ahmad H., Habumugisha J.M., Washakh R.M.A., Alam M., Liu E. (2021). Flooding and Its Relationship with Land Cover Change, Population Growth, and Road Density. Geosci. Front..

[54] Sarkar D., Mondal P. (2020). Flood Vulnerability Mapping Using Frequency Ratio (FR) Model: A Case Study on Kulik River Basin, Indo-Bangladesh Barind Region. Appl. Water Sci..

[55] Kalantari Z., Nickman A., Lyon S.W., Olofsson B., Folkeson L. (2014). A Method for Mapping Flood Hazard along Roads. J. Environ. Manag..

[56] Thanvisitthpon N., Shrestha S., Pal I., Ninsawat S., Chaowiwat W. (2020). Assessment of Flood Adaptive Capacity of Urban Areas in Thailand. Environ. Impact Assess. Rev..

[57] Moghadas M., Asadzadeh A., Vafeidis A., Fekete A., Kötter T. (2019). A Multi-Criteria Approach for Assessing Urban Flood Resilience in Tehran, Iran. Int. J. Disaster Risk Reduct..

[58] Li P., Sheng M., Yang D., Tang L. (2019). Evaluating Flood Regulation Ecosystem Services under Climate, Vegetation and Reservoir Influences. Ecol. Indic..

[59] Sheng F., Liu S., Zhang T., Liu G., Liu Z. (2022). Quantitative Assessment of the Impact of Precipitation and Vegetation Variation on Flooding under Discrete and Continuous Rainstorm Conditions. Ecol. Indic..

[60] Luo Y., Yang Y., Yang D., Zhang S. (2020). Quantifying the Impact of Vegetation Changes on Global Terrestrial Runoff Using the Budyko Framework. J. Hydrol..

[61] Li P., Yang D., Chaubey I., Su X., Jiang Y. (2022). Evolution of the Freshwater Provisioning Services under Climate Change and Vegetation Restoration Influences. Ecol. Indic..

[62] Sabrin S., Karimi M., Nazari R., Pratt J., Bryk J. (2021). Effects of Different Urban-Vegetation Morphology on the Canopy-Level Thermal Comfort and the Cooling Benefits of Shade Trees: Case-Study in Philadelphia. Sustain. Cities Soc..

[63] Kaur R., Gupta K. (2022). Blue-Green Infrastructure (BGI) Network in Urban Areas for Sustainable Storm Water Management: A Geospatial Approach. City Environ. Interact..

[64] Chen W., Wang W., Huang G., Wang Z., Lai C., Yang Z. (2021). The Capacity of Grey Infrastructure in Urban Flood Management: A Comprehensive Analysis of Grey Infrastructure and the Green-Grey Approach. Int. J. Disaster Risk Reduct..

[65] García Sánchez F., Govindarajulu D. (2023). Integrating Blue-Green Infrastructure in Urban Planning for Climate Adaptation: Lessons from Chennai and Kochi, India. Land use policy.

[66] Battemarco B.P., Tardin-Coelho R., Veról A.P., de Sousa M.M., da Fontoura C.V.T., Figueiredo-Cunha J., Barbedo J.M.R., Miguez M.G. (2022). Water Dynamics and Blue-Green Infrastructure (BGI): Towards Risk Management and Strategic Spatial Planning Guidelines. J. Clean. Prod..

[67] Tanaka T., Kiyohara K., Tachikawa Y. (2020). Comparison of Fluvial and Pluvial Flood Risk Curves in Urban Cities Derived from a Large Ensemble Climate Simulation Dataset: A Case Study in Nagoya, Japan. J. Hydrol..

[68] Lin W., Sun Y., Nijhuis S., Wang Z. (2020). Scenario-Based Flood Risk Assessment for Urbanizing Deltas Using Future Land-Use Simulation (FLUS): Guangzhou Metropolitan Area as a Case Study. Sci. Total Environ..

[69] Pallathadka A., Sauer J., Chang H., Grimm N.B. (2022). Full Title: Urban Flood Risk and Green Infrastructure: Who Is Exposed to Risk and Who Benefits from Investment? A Case Study of Three U.S. Cities. Landsc. Urban Plan..

[70] Zhang K., Shalehy M.H., Ezaz G.T., Chakraborty A., Mohib K.M., Liu L. (2022). An Integrated Flood Risk Assessment Approach Based on Coupled Hydrological-Hydraulic Modeling and Bottom-up Hazard Vulnerability Analysis. Environ. Model. Softw..

